# Treatment of Nevus

**Published:** 1886-04-20

**Authors:** 


**Treatment of Nevus.**—W. J. Beatty (British Medical Journal) repots that he has successfully treated eight cases of nevus by the local applications of liquor arsenicalis night and morning until ulceration had taken place. He says that the cure was effected in from three to five weeks.

